# Anxiety and Motion Sickness Susceptibility May Influence the Ability to Update Orientation in the Horizontal Plane of Healthy Subjects

**DOI:** 10.3389/fnint.2021.742100

**Published:** 2021-09-14

**Authors:** Mónica Alcantara-Thome, José A. Miguel-Puga, Kathrine Jauregui-Renaud

**Affiliations:** Unidad de Investigación Médica en Otoneurología, Instituto Mexicano del Seguro Social, Ciudad de México, Mexico

**Keywords:** orientation, vestibular, anxiety, motion sickness, adults

## Abstract

Few studies have evaluated the influence of idiosyncrasies that may influence the judgment of space-time orientation after passive motion. We designed a study to assess the influence of anxiety/depression (which may distort time perception), motion sickness susceptibility (which has been related to vestibular function, disorientation, and to the velocity storage mechanism), and personal habits on the ability to update orientation, after passive rotations in the horizontal plane. Eighty-one healthy adults (22–64 years old) accepted to participate. After they completed an in-house general health/habits questionnaire, the short Motion Sickness Susceptibility Questionnaire, the Hospital Anxiety and Depression Scale (HADS), the Pittsburgh Sleep Quality Index, and the short International Physical Activity Questionnaire, they were exposed to 10 manually driven whole-body rotations (45°, 90°, or 135°), in a square room, with distinctive features on the walls, while seated in the normal upright position, unrestrained, with noise-attenuating headphones and blindfolded. After each rotation, they were asked to report which wall or corner they were facing. To calculate the error of estimation of orientation, the perceived rotation was subtracted from the actual rotation. Multivariate analysis showed that the estimation error of the first rotation was strongly related to the results of the orientation test. The magnitude and the frequency of estimation errors of orientation were independently related to HADS anxiety sub-score and to adult motion sickness susceptibility, with no influence of age, but a contribution from the interaction of the use of spectacles, the quality of sleep and sex. The results suggest that idiosyncrasies may contribute to the space-time estimation of passive self-motion, with influence from emotional traits, adult motion sickness susceptibility, experience, and possibly sleep quality.

## Introduction

Behavior in the environment requires a dynamic updating of the perceptions of the body and the surroundings of the body. The spatial updating of self-to-object directions and distances (egocentric relations) that takes place concurrently with the change of spatial relations independent of the position of the perceiver (allocentric relations) depends upon the availability of multisensory information (Klatzky, 1998). Evidence suggests that humans update egocentric, internalized versions of the surroundings to orient themselves as they move (Wang and Spelke, 2000). Though, from an ecological approach to perception and action (Gibson, 1966), perception may not be based on patterns of stimulation available to individual perceptual systems, but may take advantage of “higher order relations” between them (Stoffregen and Riccio, 1988).

During active movements, comparison between the internal prediction of the proprioceptive consequences of self-motion to the actual resultant feedback, input carried by vestibular afferents may be canceled in conditions where there is a match between predicted and actual proprioceptive feedback (Cullen, 2011). Neurons in the vestibular nuclei differentially encode active versus passive head motion; during active movements, distinct classes of neurons allow for reduction of vestibular signals in order to redirect gaze or to stabilize head in space (for review Cullen and Roy, 2004). However, it is unknown yet how this differential processing of head velocity at the vestibular nucleus contributes to other vestibular functions.

In the absence of vision, vestibular signals may update self-orientation in the environment (Crum-Brown, 1875; Mach, 1875). Throughout passive motion, perception of displacement is derived from the same signal that determines perception of velocity, by integration over time (Mergner et al., 1996); while space-time relativity seems to be independent of whether velocity, distance derived by path integration, or both variables are stored in spatial working memory (Glasauer et al., 2007). Besides, vestibular perception of passive rotation in the horizontal plane can be independent of whether subjects are standing or sitting during rotation (Becker et al., 2000); while moving on to active behavior, displacement perception may be modified by proprioceptive and efferent signals, as well as the vestibular afferents at the pace of stepping (Jürgens et al., 1999).

After whole body passive rotations around an earth-vertical axis, without visual cues, subjects can indicate their orientation in space with respect to their initial orientation, while they update their actual orientation with respect to the surroundings (Israël et al., 1996; Wang and Spelke, 2000; Jáuregui-Renaud et al., 2008). Using simultaneous measurement of oculo-motor and perceptual measures of the vestibular time constant has shown that the perception of angular velocity is based on signals subserved by the velocity storage mechanism (Okada et al., 1999). This mechanism lengthens the time constant of the oculo-motor response to constant head rotation when no vision is available (Raphan et al., 1979), in such a way that the vestibulo-ocular response and the perception of self-motion outlast the duration of the response from the semicircular canals (Grunfeld et al., 2000; Bertolini et al., 2012).

The velocity storage mechanism has also been related to motion sickness susceptibility (for review Cohen et al., 2019). However, there are many theories of motion sickness, and some reject explanations in terms of velocity storage (Stoffregen and Riccio, 1988).

In addition, during passive rotation in the dark, updating orientation is dependent on vestibular inputs and processing in the central nervous system, with no opportunity to anticipate motion (for review see Behrendt, 2013; Eichenbaum, 2017). Then, both unpredictability and uncertainty may enable emotional responses during updating orientation in the dark (for review see Lake and LaBar, 2011);

The accuracy to judge angular displacements is highly variable among healthy subjects (Guedry et al., 1971), and overestimation of rotation is more frequent than underestimation (Israël et al., 1995; Marlinsky, 1999; Jáuregui-Renaud et al., 2008; Anson et al., 2021). It may decrease in old age (Anson et al., 2021), but other individual factors are poorly documented. We designed a study to assess the influence of individual factors on the vestibular contribution to update orientation, after passive rotations in the horizontal plane in young and middle-aged adults.

We selected the following factors that may influence time perception or space-time perception, as well as personal habits that could influence performance: common mental symptoms (anxiety/depression), which have been related to distortions on the awareness of time (Droit-Volet, 2013); susceptibility to motion sickness (in cars, boats, planes, trains, funfair rides), which is related to vestibular function (for review Money, 1970), to disorientation (for review Yardley, 1991), and which could be related to the velocity storage mechanism (for review Cohen et al., 2019); as well as physical activity (Rogge et al., 2021), quality of sleep (Martínez-Gallardo et al., 2020), the use of spectacles (Demer and Crane, 1998), and alcohol/tobacco use (Hafström et al., 2007).

## Materials and Methods

### Participants

After approval by the Research and Ethics Committees, 81 subjects (mean age 40.0 years ± standard deviation 11.0 years) accepted to participate. All the participants denied having a history of dizziness, vertigo, unsteadiness, migraine, hearing loss, and neurological or psychiatric disorders (submission to psychiatric care of psychopharmacological treatment); none of them had evidence of vestibular dysfunction assessed by neuro-otology evaluation with caloric/rotational tests, and all of them were naive to the study protocol and to the orientation test.

Once they have completed questionnaires to assess the individual factors, they performed an updating orientation test.

### Questionnaires

An in-house questionnaire of general health and personal habits.

The Hospital Anxiety and Depression Scale (HADS) (Zigmond and Snaith, 1983), which is a self−report screening scale that contains 14−items with a Likert scale, seven for anxiety and seven for depression. It has been used to identify possible and probable cases of anxiety disorders in samples from the general population, general practice and psychiatric patients (Bjelland et al., 2002). It is scored by summing the ratings for all the items to yield a total score, and by summing the ratings for the seven items of each subscale to yield two separate sub-scores, which range from 0 to 21. A cut-off score of ≥8 for both subscales gives sensitivities and specificities in the range of 0.70–0.90 (Zigmond and Snaith, 1983; Bjelland et al., 2002), and Cronbach’s alpha coefficient varying from 0.67 to 0.93 (Bjelland et al., 2002).

The short from of the Motion Sickness Susceptibility Questionnaire (Golding, 2006b), which is a self−report screening scale that contains 18−items, divided into two parts: part A assessing motion sickness during childhood and part B assessing motion sickness during adulthood. It predicts individual differences in motion sickness caused by a variety of stimuli (e.g., cars, boats, planes, trains, funfair rides). Each score ranges from 0 (no susceptibility) to 27 (maximal level of susceptibility) and gives a total score from 0 to 54, with a Cronbach’s alpha coefficient of 0.87 (Golding, 2006b).

The Pittsburgh Sleep Quality Index (Buysse et al., 1989), which is a self-rated questionnaire to assess sleep quality and sleep disturbances. Nineteen items generate seven scores on: subjective sleep quality, sleep latency, sleep duration, habitual sleep efficiency, sleep disturbances, use of sleeping medication, and daytime dysfunction. The combination of these sub-scores also generates three separate factors to assess (Jia et al., 2019): sleep efficiency; sleep latency and sleep quality. A total score >5 can be considered as bad quality of sleep (Buysse et al., 1989), with Cronbach’s alpha coefficient varying from 0.70 to 0.83 (Mollayeva et al., 2016).

The short form of the International Physical Activity Questionnaire (IPAQ) (Craig et al., 2003), which is a self-report instrument to assess the frequency and duration of vigorous, moderate and walking activities, as well as the average sitting time on a weekday during the last 7 days. Although the overall scale tends to overestimate the amount of physical activity, it has shown acceptable correlations with objective measures of activity to assess walking (Lee et al., 2011).

### Updating Orientation Test

To assess self-orientation relative to the distinctive features of an unfamiliar room, participants were seated in the normal upright position, unrestrained on a hydraulic barber’s chair (with a gyroscope on the headrest), in the center of a squared room (2 × 2 m), which contained fixed features positioned in the middle of each wall, in such a way that the features and corners subtended 45° with respect to the subject. They were asked to remember the location of each of the features while they were rotated in the light, and faced each of the walls. Then, blindfolded and wearing noise-attenuating headphones, they were exposed to two sets of five manually driven whole body rotations of 45° (2 s) or 90° (3 s) or 135° (4 s), to the right or to the left, in an unpredictable sequence, balanced for amplitude, direction, and order, with 10 s in-between (to allow post-rotatory sensations to fade). After the first set of five rotations and return to the start position, the eye mask was removed and the subject had a short rest of 1–2 min before commencing the second set of rotations. The actual rotation sequence included five rotations to the right and five rotations to the left (four rotations of 45°, five rotations of 90°, and 1 rotation of 135°); the sequence of small and large rotations and vice versa were similar to the right than to the left; whenever the first rotation of the first set was to the right, then the first rotation of the second set (after the rest) was to the left, and vice versa. Participants were instructed to report which wall or corner they were facing at the moment, just after each rotation (Jáuregui-Renaud et al., 2008).

The estimation error of each rotation was computed by subtracting the reported rotation from the actual rotation. Average estimation errors were calculated for all rotations and for each rotation size (error magnitude), and the number of correct estimations (error frequency) was also considered. All participants were tested during the morning and early afternoon.

### Analysis

Statistical analysis was performed using STATISTICA software (Tulsa, OK., StatSoft Inc.). According to data distribution (Kolmogorov Smirnov test), the results are described using median and quartiles 1 and 3 (Q1–Q3). The exploratory bivariate analysis was performed using Pearson correlation coefficient to assess linear correlations, and Mann Whitney *U* test to compare subgroups by age (≤ or >40 years old), by sex, and by HADS anxiety sub-score ≥8. To further assess linear correlations, analysis of covariance was performed. Considering the data distribution and plausible not linear effects, to assess the contribution of each independent variable to predict the estimation errors and correct estimations, after controlling for all other independent variables, a multivariable analysis was performed using a Generalized Linear Model with Wald test (Lindsey, 1997). The significance level was set at 2-tailed 0.05.

## Results

### General Description

The characteristics of the participants and the scores on the Motion Sickness Questionnaire and the HADS are described in Table 1. The majority of participants was right-handed and had a university degree; circa half of them were health workers and a third of them were administrative workers. The report of physical activity was varied: 27.1% (*n* = 22) reported low physical activity, 29.6% (*n* = 24) reported moderate physical activity and 43.2% (*n* = 35) reported high physical activity. The use of alcohol was frequent but moderate (53%, *n* = 43) as well as the report of bad quality of sleep (Pittsburgh Sleep Quality Index >5) (51%, *n* = 42).

**TABLE 1:** General characteristics and questionnaire scores of the 81 adults participating in the study.

**Variables**	**Percentage (number)**
Handness	
Right	86.4% (70)
Left	4.9% (4)
Ambidextrous	8.6% (7)
Education	
High school	2.4% (2)
College	28.3% (23)
University graduate	46.9% (38)
University post-graduate	12.3% (10)
Occupation	
Health workers	56.7% (46)
Administrative workers	33.3% (27)
Other occupation	8.6% (7)
Tobacco and alcohol	
Tobacco smokers	11.1% (9)
Alcohol use	53.0% (43)
Corrected Myopia/Astigmatism	35.8% (29)
	**Median (Q1–Q3)**
Pittsburgh sleep quality index	
Sleep Efficiency	2 (1–3)
Sleep Latency	1 (0–2)
Sleep Quality	3 (2–4)
Total score	6 (3–8)
International Physical Activity Questionnaire (met-minutes per week)	
Vigorous activity	240 (0–1200)
Moderate activity	120 (0–720)
Walking	495 (157–1287)
Total activity	1386 (480–3576)
Hospital Anxiety and Depression Scale	
Anxiety score	3 (1–7)
Depression score	1 (0–3)
Total score	5 (2–10)
Motion sickness susceptibility questionnaire	
Before age 12	2 (0–4.5)
Over the last 10 years	1 (0–3)
Total score	3.6 (0–7.5)
Updating Orientation Test	
Estimation error	
First set of rotations	18° (9°–27°)
Second set of rotations	18° (9°–27°)
All rotations	18° (9°–22.5°)
45° rotations	11.2° (0°–22.5°)
90° rotations	18° (9°–27°)
135° rotation	0° (0°–45°)
Total Correct Estimations	7 (5–8)

The results of the orientation test are shown in Table 1. Inaccurate estimation of orientation was observed after 34.3% of all rotations, with a typical error of 45°; overestimation of rotation was more frequent than underestimation of rotation (85.3% versus 14.7%). The median estimation error for all rotations was 18° (Q1–Q3 = 9°–22.5°); it was the same for the two sets of rotations (18°, 9°–27°), and it was consistent with the median estimation error for the 90° rotations (18°, 9°–27°). The median of the total correct estimations was 7 (5–8).

### Exploratory Bivariate Analysis

Comparisons by age are shown in Table 2. Compared to young adults (21–40 years old), middle-aged participants (41–64 years old) showed the lowest scores on the Motion Sickness Susceptibility Questionnaire total score and sub-scores (*p* ≤ 0.008), and the highest scores on the Pittsburgh Quality of Sleep Index total score and sub-scores (*p* ≤ 0.03), with no differences between subgroups on the average estimation error or the correct estimations.

**TABLE 2:** Median, Quartile 1 (Q1) and Quartile 3 (Q3) of the Pittsburgh sleep quality index, International Physical Activity Questionnaire, Hospital Anxiety and Depression Scale, Motion Sickness Susceptibility Questionnaire, and updating orientation results by age (young adults ≤40 years *versus* middle age >40 years).

**Comparisons by age group**				
	
	**≤40 years (*n* = 42) Median (Q1–Q3)**	**>40 years (*n* = 39) Median (Q1–Q3)**	***Z* value**	**Bilateral *p* value**
Pittsburgh sleep quality index				
Sleep Efficiency	1 (1–2)	2 (1–3)	−2.216	**0.030**
Sleep Latency	1 (0–2)	1 (0–2)	−0.325	0.759
Sleep Quality	3 (2–3)	3 (2–5)	−2.275	**0.025**
Total score	5 (3–7)	6 (4–9)	−2.270	**0.023**
International Physical Activity Questionnaire (met-minutes per week)				
Vigorous activity	960 (0–1920)	80 (0–960)	1.377	0.191
Moderate activity	360 (0–840)	0 (0–720)	1.685	0.113
Walking	693 (198–1386)	380 (99–693)	1.707	0.089
Total activity	2492 (678–4095)	990 (405–3135)	1.722	0.086
Hospital Anxiety and Depression Scale				
Anxiety score	3.5 (1–6)	3 (0–7)	0.418	0.682
Depression score	1 (0–2)	1 (0–3)	−0.616	0.556
Total score	4.5 (2–9)	5 (1–11)	0.123	0.906
Motion sickness susceptibility questionnaire				
As a child (before age 12)	3 (1.1–5.1)	1.1 (0–4)	2.635	**0.008**
Over the last 10 years	2 (0–3.3)	0 (0–1.8)	2.924	**0.004**
Total score	4.75 (2–9)	2 (0–5.5)	2.964	**0.002**
Updating Orientation Test				
Average estimation error				
First set of rotations	18° (9°–27°)	18° (9°–27°)	0.587	0.575
Second set of rotations	18° (9°–27°)	18° (9°–27°)	0.222	0.832
All rotations	18° (9°–22.5°)	13.5° (9°–27°)	0.497	0.628
45° rotations	11.2° (11.2°–22.5°)	11.2° (0°–22.5°)	−0.468	0.655
90° rotations	18° (9°–27°)	18° (9°–18°)	0.722	0.489
135° rotation	22.5 (0°–45°)	0° (0°–45°)	0.179	0.876
Total correct estimations	7 (5–8)	7 (5–8)	−0.311	0.759

*Comparisons were performed using Mann-Whitney *U* test. Significant values are highlighted in bold.*

Comparisons by sex are shown in Table 3. Although men spent more energy on physical activity (met-minutes per week) than women (*p* = 0.021), there were no other differences by sex.

**TABLE 3:** Median, Quartile 1 (Q1) and Quartile 3 (Q3) of the Pittsburgh sleep quality index, International Physical Activity Questionnaire, Hospital Anxiety and Depression Scale, Motion Sickness Susceptibility Questionnaire, and updating orientation results by sex.

**Comparisons by sex**				
	
	**Women (*n* = 52) Median (Q1–Q3)**	**Men (*n* = 29) Median (Q1–Q3)**	***Z* value**	**Bilateral *p* value**
Pittsburgh sleep quality index				
Sleep Efficiency	2 (1–3)	1 (1–2)	1.675	0.105
Sleep Latency	1 (0–2)	1 (1–2)	–1.663	0.111
Sleep Quality	3 (2–4.5)	3 (1–4)	0.517	0.613
Total score	6 (3–8.5)	6 (3–7)	0.460	0.648
International Physical Activity Questionnaire (met-minutes per week)				
Vigorous activity	0 (0–1200)	960 (0–2000)	–1.988	0.057
Moderate activity	0 (0–540)	240 (0–1200)	–1.877	0.076
Walking	495 (149–990)	693 (165–1386)	–0.814	0.419
Total activity	1056 (396–3247)	2506 (809–6008)	–2.298	**0.021**
Hospital Anxiety and Depression Scale				
Anxiety score	4 (1–7)	2 (0–5)	1.538	0.128
Depression score	2 (0–3)	0 (0–2)	1.465	0.161
Total score	5 (2–10.5)	3 (0–7)	1.706	0.089
Motion sickness susceptibility questionnaire				
As a child (before age 12)	2 (0–4.5)	2 (0–5.1)	–0.354	0.728
Over the last 10 years	1.1 (0–3.1)	1 (0–2)	0.745	0.477
Total score	3.3 (0.5–8.2)	3.6 (1.1–6)	–0.049	0.964
Updating Orientation Test				
Estimation error				
1st set of rotations	18° (9°–27°)	18° (9°–18°)	0.805	0.441
2nd set of rotations	18° (9°–27°)	18° (9°–18°)	0.615	0.553
All rotations	18° (9°–27°)	13.5° (9°–22.5°)	0.872	0.390
45° rotations	11.2° (0°–22.5°)	11.2° (11.2°–22.5°)	–0.241	0.818
90° rotations	18° (9°–27°)	9° (9°–18°)	1.027	0.324
135° rotation	22.5° (0°–45°)	0° (0°–45°)	0.681	0.546
Total correct estimations	7 (5–8.1)	7 (6–8)	–0.648	0.527

*Comparisons were performed using Mann-Whitney *U* test. Significant values are highlighted in bold.*

Comparison by HADS anxiety sub-score showed that participants with a sub-score ≥8 reported fewer accurate estimations (*Z* = -2.013, *p* = 0.044) and larger overestimation of rotation (*Z* = 1.985, *p* = 0.047) than those with a sub-score <8.

Weak linear correlations were observed between the results of the orientation test and the HADS score and sub-scores (anxiety sub-score and depression sub-score) (Pearson’s *r* < 0.3, *p* < 0.05), as well as the motion sickness total score and adult sub-score (Pearson’s *r* < 0.3, *p* < 0.05) (Appendix 1). The strongest linear correlations were observed between the estimation error of the first rotation and the average estimation error, the total correct estimations and the estimation error for the 90° rotations (Pearson’s r from 0.48 to 0.67, *p* < 0.00001) (Appendix 1).

**FIGURE 1:**
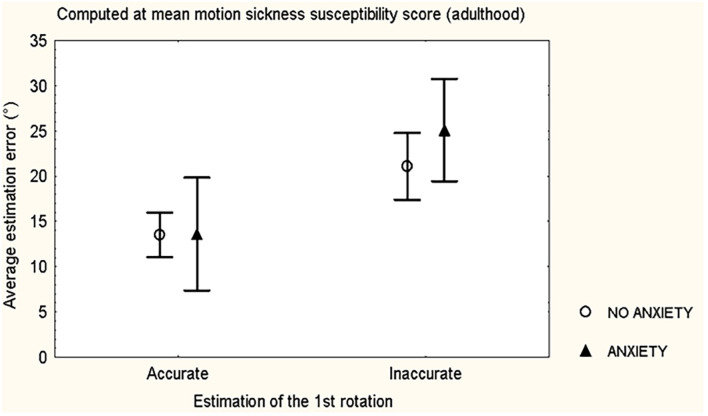
Mean and standard error of the mean of the average estimation error during the updating orientation test, according to accuracy/inaccuracy of the estimation of the first rotation and evidence of Anxiety/No anxiety (HADS anxiety sub-score ≥8), of 81 healthy volunteers.

Further covariance analysis confirmed that inaccurate estimation of the first rotation was linearly related to increased average estimation error, independently from HADS anxiety sub-score ≥8 and from the motion sickness susceptibility adult sub-score (*F* = 15.630, *p* = 0.0001) (Figure 1); while no significant linear interaction between these two variables was observed (*F* = 0.672, *p* = 0.4).

### Multivariable Analysis

The Estimates with their Standard Error and the Wald statistic with *p* values for all the variables included in the model are shown in Table 4A. The anxiety sub-score of the HADS and the adult sub-score of the Motion Sickness Susceptibility Questionnaire had independent contributions to both the average estimation error (*p* ≤ 0.009), and the total correct estimations (*p* ≤ 0.023), with a contribution of the use of spectacles on the total correct estimations (*p* ≤ 0.035), but no influence was observed from age, physical activity, smoking, or alcohol use. In addition, in men, the use of spectacles and bad quality of sleep was related to larger average estimation error (*p* = 0.010) and less total correct estimations (*p* = 0.0004).

**TABLE 4:** Coefficient estimates and standard error (S.E.) of the estimates are described with Wald statistic and *p* values for the average estimation error and the total correct estimations without including the first rotation estimation error **(A)** and including it **(B)**.

	Estimation error *Estimate ± S.E.*	Correct estimations *Estimate ± S.E.*
**A**		

Intercept	2.410 ± 0.121	2.057 ± 0.039
Wald Statistic (*p* value)	396.890 (**<0.0001**)	2650.439 (**<0.0001**)
Adult Motion sickness score	0.054 ± 0.019	–0.030 ± 0.013
Wald Statistic (*p* value)	7.744 (**0.0005**)	5.096 (**0.023**)
HADS Anxiety score	0.0290 ± 0.011	–0.018 ± 0.007
Wald Statistic (*p* value)	6.654 (**0.009**)	7.194 (**0.007**)
Sex	0.082 ± 0.095	–0.009 ± 0.027
Wald Statistic (*p* value)	0.741 (0.389)	0.1344 (0.713)
Use of spectacles	0.173 ± 0.095	–0.057 ± 0.027
Wald Statistic (*p* value)	3.318 (0.068)	4.400 (**0.035**)
Quality of sleep	0.107 ± 0.097	–0.050 0.028
Wald Statistic (*p* value)	1.237 (0.265)	3.134 (0.076)
Sex * Use of spectacles * Quality of Sleep	0.245 ± 0.095	–0.094 ± 0.027
Wald Statistic (*p* value)	6.533 (**0.010**)	12.202 (**0.0004**)

**B**		

Intercept	2.351 ± 0.110	2.091 ± 0.037
Wald Statistic (*p* value)	451.183 (**<0.0001**)	3160.707 (**<0.0001**)
Adult Motion sickness score	0.025 ± 0.018	–0.026 ± 0.012
Wald Statistic (*p* value)	1.871 (0.171)	4.290 (**0.038**)
HADS Anxiety score	0.0233 ± 0.010	–0.0121 ± 0.006
Wald Statistic (*p* value)	5.317 (**0.021**)	3.464 (0.062)
1st Rotation Estimation error	0.009 ± 0.002	–0.005 ± 0.001
Wald Statistic (*p* value)	15.496 (**<0.0001**)	17.717 (**<0.0001**)
Sex	0.045 ± 0.086	0.012 ± 0.024
Wald Statistic (*p* value)	0.276 (0.598)	0.268 (0.604)
Use of spectacles	0.134 ± 0.085	–0.046 ± 0.024
Wald Statistic (*p* value)	2.475 (0.115)	3.533 (0.060)
Quality of sleep	0.064 ± 0.087	–0.024 ± 0.026
Wald Statistic (*p* value)	0.536 (0.463)	0.852 (0.355)
Sex * Use of spectacles * Quality of Sleep	0.235 ± 0.086	–0.090 ± 0.024
Wald Statistic (*p* value)	7.480 (**0.006**)	14.036 (**0.0001**)

*Significant values are highlighted in bold.*

To confirm the independent contribution of the estimation error of the first rotation to the overall results of the orientation test, including plausible no linear effects, a second multivariable analysis was performed including this variable (despite collinearity). The Estimates with their Standard Error and the Wald statistic with *p* values for all the variables included in the model are shown in Table 4B. A highly significant relationship was observed between the estimation of the first rotation and the overall results of the test (*p* < 0.0001); participants who made an accurate estimation of the first rotation had lower average estimation error than those who made an inaccurate first estimation.

## Discussion

In young and middle-aged healthy subjects, assessment of the influence of individual factors on updating orientation during passive rotations in the horizontal plane showed contributions from anxiety and adult motion sickness susceptibility (in cars, boats, planes, trains, funfair rides), as well as an interaction among the use of spectacles, the quality of sleep and sex, with no independent influence from age or sex. Estimation of the first rotation was related to the accuracy in updating orientation during the following rotations.

The finding of an association between HADS anxiety sub-score and the results of the orientation test is consistent with the relationship between emotion and vestibular function (Balaban and Thayer, 2001; Viaud-Delmon et al., 2011; Preuss et al., 2014; Coelho and Balaban, 2015). The spectrum of this relationship comprises from the dizziness related to psychiatric disorders (for review see Viaud-Delmon et al., 2011) to the neural network of vestibular inputs (Balaban and Thayer, 2001; Balaban, 2002). However, in this study we also considered the influence of affective states on the subjective estimation of time (Pariyadath and Eagleman, 2007; Tanaka and Yotsumoto, 2017). Since anxiety has been related to distortions of the awareness of time, including overestimation of short time intervals (for review see Droit-Volet, 2013).

In agreement with previous reports using the same or a similar orientation task (Israël et al., 1995; Marlinsky, 1999; Jáuregui-Renaud et al., 2008; Anson et al., 2021), in this study, over-estimation of rotation was more frequent than underestimation. The orientation test design included rotations of increasing amplitude/duration, this allowed overestimation of duration to be easily interpreted as increased amplitude, which could give rise to overestimation errors. Participants with a HADS anxiety sub-score ≥8 were prone to overestimate the rotations. We propose the hypothesis that distortion of the duration of rotation could have influenced the percept of displacement. However, variation of the velocity among rotations (11°/s) could have interfered with this result.

The association of motion sickness susceptibility (in cars, boats, planes, trains, funfair rides), with errors on updating orientation suggests that the unknown idiosyncrasy related to the variability of motion sickness susceptibility among subjects (Lentz and GuedryJr., 1978; Golding, 2006a) may also contribute to distortions on the space-time perception of passive rotations. We suggest that a link between these two variables could be the velocity storage mechanism and its processing, by means of the source signal to perceive both displacement and duration of rotation. The finding of lower motion sickness susceptibility scores in participants older than 40 years is consistent with the report of its decline with age, in both healthy subjects and vestibular patients (Paillard et al., 2013; Jones et al., 2019).

The independent contribution of motion sickness susceptibility and trait anxiety (by HADS anxiety sub-score) to the estimation error of orientation is in agreement with the report that the relationship between trait anxiety and motion sickness susceptibility can be weak in healthy subjects and not evident in patients with vestibular disease (Paillard et al., 2013).

The relationship between the estimation accuracy observed during the first rotation of the orientation test and the performance during the whole test could be explained by the test itself. Since participants were blindfolded, and they had no opportunity to contrast or reset their space-time perception of rotation with other sensory cues, they had to rely on their vestibular inputs and use the memory trace of their initial motion perception as they rotated to face each new direction. In addition, the strongest correlation with the estimation error of the 90° rotations could have been influenced by the reference axis. During passive rotations without vision, evidence suggests that the spatial framework used to organize the egocentric space orientation may include an orthogonal system related to the frame of the human body, with an ideal angle of 90° for subjective estimations of turns in the range of 15°–165° (Sadalla and Montello, 1989).

Several factors may have contributed to the finding that the use of spectacles and bad quality of sleep were related to larger average estimation error and less correct estimations, particularly in men. In healthy subjects, visual conditions can modify the vestibular responses (Gonshor and Jones, 1976). In laboratory situations, including passive head rotation, the wearing of telescopic spectacles can modify the gain of the vestibulo-ocular reflex (Demer and Crane, 1998). However, an objective evaluation of the corrected refraction errors would be required to adequately assess this factor. On the other hand, sleep disturbances have been related to several deficits including attention, memory, cognitive processes and emotional reactivity (for review see McCoy and Strecker, 2011); likewise, they can alter vestibular responses (Collins, 1988; Quarck et al., 2006; Martin et al., 2018) and they can affect the perception of motion during magnetic vestibular stimulation (Martínez-Gallardo et al., 2020). Evidence supports that, after sleep deprivation, the vestibulo-ocular reflex gain may increase in response to velocity steps (Quarck et al., 2006), or the responses may decrease according to the time of sleep deprivation (Collins, 1988). Moreover, in this study, since participants were blindfolded, we cannot disregard the effect of darkness on the alertness of subjects with bad quality of sleep (de Zeeuw et al., 2019). In addition, recent evidence suggests that sleep deprivation could have a stronger effect on balance (i.e., postural stability) in men, compared to women (Ołpińska-Lischka et al., 2021).

In agreement with previous reports (Jáuregui-Renaud et al., 2008; Anson et al., 2021), the analysis of this study showed no independent influence of sex on updating orientation after passive rotations in the horizontal plane. This finding was consistent with the observed lack of influence of physical activity on the performance of the orientation test, given that men reported more physical activity than women. Previous reports support that men may out-perform women on virtual navigation tasks (Astur et al., 1998) and way-finding outdoors (Silverman et al., 2000). However, men may prefer an allocentric strategy to orient them-selves (Dabbs et al., 1998), while women may prefer a landmark-based strategy (Choi et al., 2006). Albeit the orientation test of this study implied a combination of egocentric and allocentric strategies, an egocentric framework may have been used to update object position while rotating. This is consistent with the evidence supporting that the ability to accurately locate objects in small-scale environments appears to depend on the perception of the current egocentric distances and directions of objects, with a continuous update of these perceptions relative to the environment geometry through time, which can be used as a source for reorienting over motion (for review see Cheng et al., 2013), while path integration using the head as a reference is egocentric by nature. Moreover, in this study, the reported localization was not by chance; which supports that egocentric spatial perceptions persisted over orientation/disorientation.

We also observed no significant correlation between the age of the participants and the average estimation error or the total correct estimations. Young and middle-aged adults showed similar results on the orientation test, despite clear differences on the motion sickness susceptibility scores. These findings support that the decline in spatial orientation that has been observed in the elderly could be attributed to factors that may develop late in life (Anson et al., 2021), but may not be evident yet in young and middle-aged subjects (Jáuregui-Renaud et al., 2008).

**FIGURE 2:**
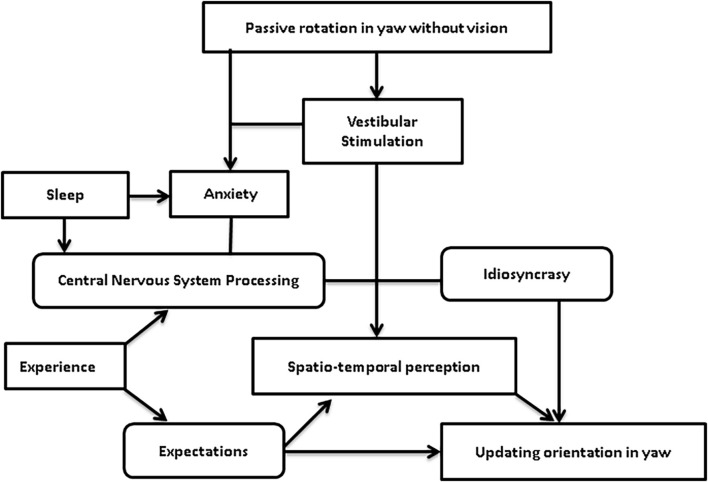
Theoretical model of the interaction among individual factors during passive rotation without vision to update orientation in the horizontal plane.

An interaction among these variables is represented in Figure 2. The results suggest that updating orientation in the horizontal plane during passive rotations, without vision, would be the result of the space-time perception of the stimuli, in the context of individual expectations and idiosyncrasy. However, to estimate both displacement and duration of motion, idiosyncrasy may play a part in central integration of the sensory inputs; while anxiety and bad sleep may influence this processing, in the context of individual experiences and expectations.

The main limitation of this study was its cross-over design. Repeated measures would allow less individual cofactors increasing the variance, and better assessment of age effects on performance; though performing a known task would have an effect on the results. A second limitation of the study is the reliance en self-report instruments; also, the apparent lack of influence of alcohol/tobacco use that is not conclusive, due to the low frequency of smokers and the moderate alcohol consumption among participants. Another limitation was that the manually driven rotations of the orientation test introduced velocity variability among the three amplitudes tested (11°/s), precluding an accurate assessment according to the space-time characteristics of the stimuli. However, the consistent findings open new research questions to study the influence of individual factors in the perception of self-motion, including the possibility that distortion of time perception might interfere with displacement perception, as well as the influence of the immediate perception of motion on updating space-time orientation.

In conclusion, in young and middle-aged healthy adults, susceptibility to motion sickness and anxiety may influence the space-time perception of earth-vertical passive rotations in the horizontal plane, with influence from individual traits and experiences, and possibly sleep quality.

## Data Availability Statement

The raw data supporting the conclusions of this article will be made available by the authors, without undue reservation.

## Ethics Statement

The studies involving human participants were reviewed and approved by Comité Local de Investigación en Salud y Ética en Investigación no. 3601 del Instituto Mexicano del Seguro Social. The participants provided their written informed consent to participate in this study.

## Author Contributions

KJ-R conceived and designed the study and the vestibular stimuli, supervised the selection of participants, analyzed and interpreted the data, and wrote the manuscript. MA-T selected and evaluated the participants, performed the stimuli, collected the data, and revised the manuscript. JM-P coordinated the evaluation of participants, validated and administered the data, and revised the manuscript. All authors contributed to the article and approved the submitted version.

## Conflict of Interest

The authors declare that the research was conducted in the absence of any commercial or financial relationships that could be construed as a potential conflict of interest.

## Publisher’s Note

All claims expressed in this article are solely those of the authors and do not necessarily represent those of their affiliated organizations, or those of the publisher, the editors and the reviewers. Any product that may be evaluated in this article, or claim that may be made by its manufacturer, is not guaranteed or endorsed by the publisher.

## Appendix 1

**TABLE 1:** Correlation matrix between the updating orientation test results and the age, the first rotation estimation error, the Motion Sickness Susceptibility Questionnaire and the Hospital Anxiety and Depression Scale (HADS) total scores and sub-scores.

	**Age**	**1st rotation error**	**Motion sickness susceptibility**			**Anxiety and Depression (HADS)**		
			**Childhood**	**Adult**	**Total**	**Anxiety**	**Depression**	**Total**
Average estimation error	−0.006, *p* = 0.953	**0.486, *p* < 0.0001**	0.199, *p* = 0.076	**0.254, *p* = 0.023**	**0.244, *p* = 0.029**	**0.270, *p* = 0.015**	**0.240, *p* = 0.031**	**0.270, *p* = 0.015**
45° rotations	0.034, *p* = 0.765	0.0644, *p* = 0.570	0.107, *p* = 0.343	0.084, *p* = 0.456	0.103, *p* = 0.361	0.095, *p* = 0.399	0.121, *p* = 0.282	0.110, *p* = 0.328
90° rotations	−0.044, *p* = 0.696	**0.676, *p* < 0.0001**	0.175, *p* = 0.120	**0.233, *p* = 0.037**	**0.22, *p* = 0.047**	**0.280, *p* = 0.012**	**0.241, *p* = 0.031**	**0.278, *p* = 0.012**
135° rotation	0.006, *p* = 0.953	0.173, *p* = 0.123	0.131, *p* = 0.243	**0.235, *p* = 0.035**	0.193, *p* = 0.085	0.177, *p* = 0.115	0.115, *p* = 0.307	0.158, *p* = 0.160
Total correct estimations	−0.016, *p* = 0.887	**−0.514, *p* < 0.0001**	−0.196, *p* = 0.081	**−0.267, *p* = 0.016**	**−0.249, *p* = 0.025**	**−0.287, *p* = 0.010**	**−0.274, *p* = 0.014**	**−0.295, *p* = 0.008**

*Significant values are highlighted in bold.*
